# Supplementary material for: Saccharomyces boulardii (CNCM I-745) improves intestinal damage in sepsis by remodeling intestinal flora structure
Source: Front Cell Infect Microbiol. 2025 Jul 31;15:1602792. doi: 10.3389/fcimb.2025.1602792 (PMC12350329; doi:10.3389/fcimb.2025.1602792)
Supplement: Supplementary file 1 [file DataSheet1.zip › summary/summary/6_Taxonomy_community/1_Taxonomy_profiling/4_Order/All/Order_abund_top30.sample.cluster.pdf]

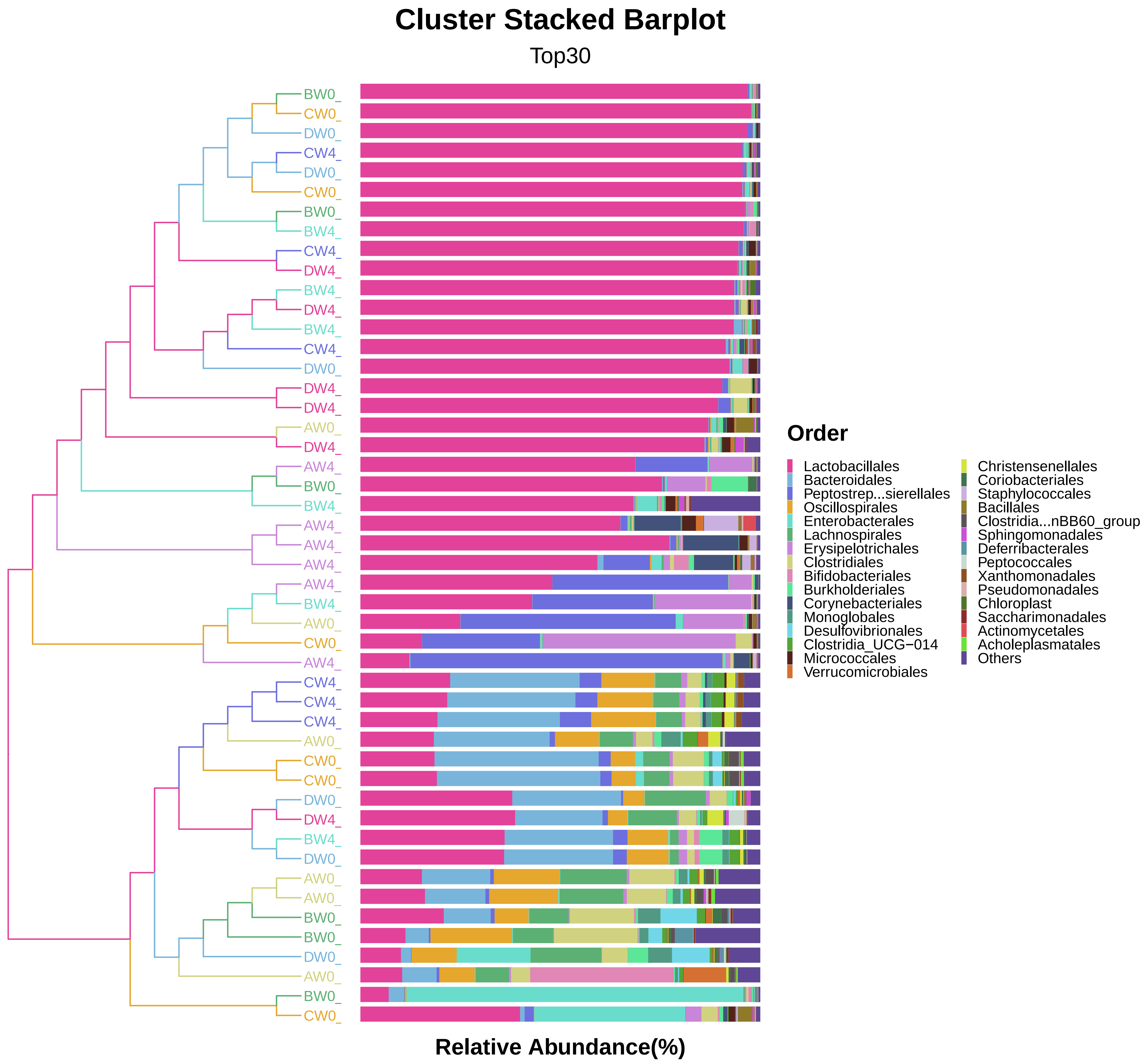
Cluster Stacked Barplot
Top30
BW0_
CW0_
DW0_
CW4_
DW0_
CW0_
BW0_
BW4_
CW4_
DW4_
BW4_
DW4_
BW4_
CW4_
DW0_
DW4_
DW4_
AW0_
DW4_
AW4_
BW0_
BW4_
AW4_
AW4_
AW4_
AW4_
BW4_
AW0_
CW0_
AW4_
CW4_
CW4_
CW4_
AW0_
CW0_
CW0_
DW0_
DW4_
BW4_
DW0_
AW0_
AW0_
BW0_
BW0_
DW0_
AW0_
BW0_
CW0_
Relative Abundance(%)
Order
Lactobacillales
Bacteroidales
Peptostrep...sierellales
Oscillospirales
Enterobacterales
Lachnospirales
Erysipelotrichales
Clostridiales
Bifidobacteriales
Burkholderiales
Corynebacteriales
Monoglobales
Desulfovibrionales
Clostridia_UCG−014
Micrococcales
Verrucomicrobiales
Christensenellales
Coriobacteriales
Staphylococcales
Bacillales
Clostridia...nBB60_group
Sphingomonadales
Deferribacterales
Peptococcales
Xanthomonadales
Pseudomonadales
Chloroplast
Saccharimonadales
Actinomycetales
Acholeplasmatales
Others
